# Pre-Analytical Cleanup of Feline Feces Improves DNA Extract Quality, Reduces Post-Extraction PCR Inhibition, and Enhances Molecular Detectability of Intestinal Protozoa

**DOI:** 10.3390/cimb48070707

**Published:** 2026-07-11

**Authors:** Dawid Jańczak, Aleksandra Kornelia Maj, Jakub Kędziorek, Mateusz Antecki

**Affiliations:** 1 Department of Infectious and Invasive Diseases and Veterinary Administration, Institute of Veterinary Medicine, Faculty of Biological and Veterinary Sciences, Nicolaus Copernicus University, Lwowska 1, 87-100 Toruń, Poland; 2 Animallab Veterinary Laboratory, Środkowa 2/4, 03-340 Warsaw, Poland; aleksandrakorneliamaj@gmail.com (A.K.M.); mateuszantecki2@gmail.com (M.A.)

**Keywords:** pre-analytical processing, fecal matrix, PCR inhibitors, DNA extraction, DNA purity, spike-recovery, post-extraction inhibition, *Tritrichomonas foetus*, *Cryptosporidium* spp., *Giardia duodenalis*, molecular parasitology

## Abstract

Feline feces represent a difficult matrix for molecular diagnostics because stool-derived inhibitors and variable sample composition may compromise DNA recovery and downstream PCR amplification. This paired methodological study evaluated whether a pre-analytical chloroform:methanol cleanup step improves DNA extract quality, post-extraction amplifiability, and molecular detection of selected intestinal protozoa in cats. A total of 105 feline fecal samples were divided into paired aliquots and subjected either to direct automated DNA extraction or to extraction preceded by chloroform:methanol cleanup. DNA concentration and purity were assessed spectrophotometrically using DNA yield, A260/280, and A260/230 ratios. Extracts from both workflows were analyzed by PCR for *Tritrichomonas foetus*, *Cryptosporidium* spp., *Pentatrichomonas hominis*, and *Giardia duodenalis*. In addition, a post-extraction spike-recovery experiment was performed on selected paired extracts to directly evaluate PCR inhibition. Cleanup significantly increased DNA concentration and improved A260/280 and A260/230 ratios. It was also associated with additional *Cryptosporidium* and *Giardia* detections and significantly lower *Cryptosporidium* Ct values among paired positives. Spike-recovery testing showed that direct extracts markedly delayed amplification of an exogenous *Cryptosporidium meleagridis* 18S spike, whereas cleanup-based extracts showed significantly lower Ct delay and higher spike recovery. All *Cryptosporidium*-positive sequences were identified as *Cryptosporidium felis*. Sequencing of the six *Giardia* beta-giardin amplicons revealed four sequences assigned to assemblage F, one sequence assigned to assemblage B, and one sequence assigned to assemblage D. These findings support chloroform:methanol cleanup as a useful pre-analytical strategy for improving feline fecal DNA extract quality and reducing post-extraction PCR inhibition, although broader validation across additional extraction platforms and larger positive sample sets is required.

## 1. Introduction

Fecal samples are one of the most challenging materials used in molecular diagnostics. The stool is a complex mixture of host-derived components, dietary residues, microbial biomass, bile-associated compounds, lipids, polysaccharides, and other substances that may negatively affect nucleic acid extraction and follow-up PCR, in contrast to biological matrices, which are generally clean. These inhibitors may reduce DNA recovery, impair DNA polymerase activity, change fluorescence signals, or generate false-negative and weakly positive results, especially when the target burden is low [1,2,3,4].

The diagnostics of feline feces make this issue particularly critical. Cats are obligate carnivores, and the composition of their feces may vary significantly depending on diet, intestinal physiology, and immune status. Recent metabolomic studies have demonstrated that the concentrations of long-chain fatty acids, sterols, and bile acid-related compounds in the feces of cats with chronic enteropathy are significantly different. This finding supports the hypothesis that feline feces can vary significantly in matrix diversity and, as a result, in their usefulness for molecular analysis [5,6].

In feline parasitology, PCR-based methods are increasingly used for the detection and characterization of enteric protozoa, including *Tritrichomonas foetus*, *Cryptosporidium* spp., and *Giardia duodenalis*. These pathogens are clinically relevant, particularly in young cats and in animals from multiple cat households. Molecular assays are usually required for confirmatory diagnosis, species identification, and genotyping [7,8,9,10,11,12,13,14]. Molecular typing is particularly important for *G. duodenalis* since animals may harbor host-adapted as well as potentially zoonotic assemblages [9,10,11]. Likewise, molecular tools improve the interpretation of *Cryptosporidium* infections, whose epidemiology and zoonotic relevance are significantly influenced by species-level identification [9,12,14].

Despite the increasing prevalence of molecular diagnostics in feline parasitology, the pre-analytical phase is still relatively underdeveloped. Previous studies have shown that fecal pre-treatment can improve the success of PCR-based detection, and inhibitor removal has been found to be a major contributor to analytical difficulties in stool testing [1,2,3,4]. However, there is limited data on whether preliminary cleanup of feline feces can improve automated DNA extraction and enhance the molecular detection of intestinal protozoa in routine veterinary laboratory procedures.

Therefore, the aim of the current study was to compare direct automated DNA extraction from feline feces with extraction preceded by a chloroform:methanol cleanup step. The comparison focused on DNA yield and spectrophotometric purity, molecular detection of selected enteric protozoa, the influence of sample-related factors such as stool consistency and color, and post-extraction PCR inhibition assessed by a spike-recovery experiment. We hypothesized that preliminary cleanup would improve DNA extract quality and molecular amplifiability by reducing matrix-associated interference, while recognizing that improved PCR performance may also reflect increased recovery of amplifiable target DNA.

## 2. Materials and Methods

### 2.1. Study Design and Samples

**Figure 1:**
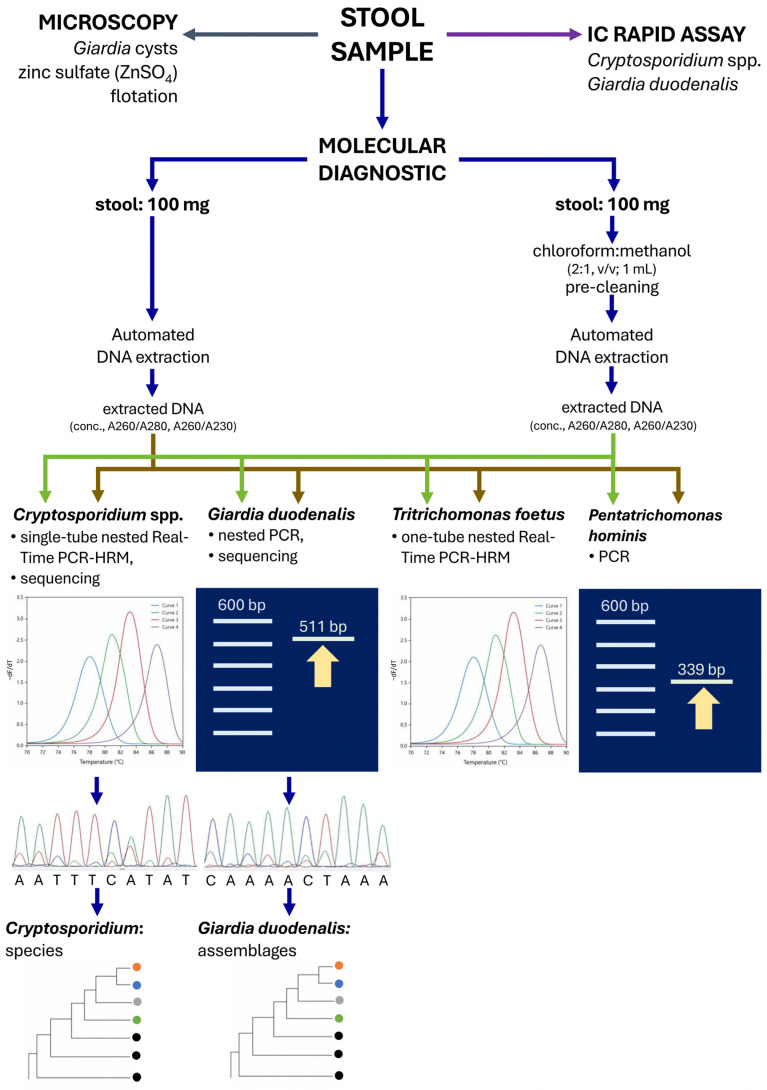
Schematic overview of the diagnostic workflow used for feline fecal samples. Each stool sample was subjected to three parallel diagnostic approaches: microscopy for *Giardia* cysts using zinc sulfate (ZnSO_4_) flotation, immunochromatographic rapid antigen testing for *Cryptosporidium* spp. and *Giardia duodenalis*, and molecular diagnostics.

The objective of this paired comparative methodological study was to determine how the molecular detection of selected intestinal protozoa in cats and the performance of automated DNA extraction are improved by preliminary fecal cleanup. Only one fecal sample from each cat was included in the analysis. After carrying out standard diagnostic testing, each fecal sample was included in the current comparative analysis. For each sample, information on age, sex, stool consistency, and stool color was recorded. Before processing, each fecal sample was thoroughly homogenized and divided into two equal aliquots of 100 mg. One aliquot was submitted directly to DNA extraction according to the manufacturer’s instructions, whereas the second aliquot underwent preliminary cleanup prior to DNA extraction (Figure 1).

### 2.2. Preliminary Cleanup Procedure

Preliminary cleanup was performed using a modified chloroform:methanol procedure based on the classical 2:1 chloroform:methanol extraction principle originally described by Folch et al. for lipid removal from biological material [15,16]. The present study’s modified protocol involved mixing 100 mg of feces with 1 mL of chloroform:methanol (2:1, *v*/*v*), vigorously mixing for 30 s, and vortexing for 10 s. After centrifugation for 5 min at 2000 rpm, the alcohol and chloroform phases were discarded, while the remaining debris was dried at 40 °C for 10 min and subsequently subjected to DNA extraction according to the manufacturer’s protocol. The paired aliquot intended for direct extraction was processed immediately without preliminary cleanup. The drying step was included to reduce residual chloroform:methanol carryover before automated DNA extraction, because organic solvent residues may interfere with extraction or PCR amplification. Residual solvent concentration was not measured analytically. Therefore, the drying procedure should be interpreted as an operational step of the tested workflow rather than as a validated solvent-removal endpoint.

### 2.3. DNA Extraction

DNA from both aliquots was extracted using the TANBead Maelstrom 4800 Nucleic Acid Extraction System (Taiwan Advanced Nanotech Inc., Taoyuan City, Taiwan, China), according to the manufacturer’s instructions. In both workflows, 100 mg of fecal material was used as the extraction matrix. The only difference between the two workflows was the presence or absence of the preliminary cleanup step before extraction. Extracted DNA was stored at −20 °C until further analyses.

### 2.4. Spectrophotometric Assessment of DNA

DNA concentration and purity were assessed spectrophotometrically using the Nano Ready Touch spectrophotometer (Hangzhou LifeReal Biotechnology Co., Ltd., Hangzhou, Zhejiang, China). For each extract, DNA concentration (ng/µL), the A260/280 ratio, and the A260/230 ratio were recorded. These parameters were used to compare the extraction performance between direct extraction and extraction preceded by preliminary fecal cleanup. The objective of this study was not only to estimate DNA concentration but also to compare extract purity between paired workflows using A260/280 and A260/230 ratios. The quantification of double-stranded DNA would be more precise through fluorometric quantification, such as Qubit-based measurement. However, absorbance-based information on protein, salt, organic compound, or other contaminant carryover would not be available.

### 2.5. PCR Detection of Tritrichomonas foetus

Detection of *Tritrichomonas foetus* was performed using an in-house one-tube nested real-time PCR-HRM assay developed as a closed-tube modification of the previously described single-tube nested PCR approach. The assay used the outer primers TFR3 (5′-CGGGTCTTCCTATAGAGACAGAACC-3′) and TFR4 (5′-CCTGCCGTTGGATCAGTTTCGTTAA-3′), together with the inner primers TFITS-F (5′-CTGCCGTTGGATCAGTTTCG-3′) and TFITS-R (5′-GCAATGTGCATTCAAAGATCG-3′). Reactions were performed in a final volume of 20 µL containing qPCR-HS Mix EvaGreen^®^, nuclease-free water, a 10× primer mix, and 2.0 µL of template DNA. Final primer concentrations were 12.5 nM for each outer primer and 0.25 µM for each inner primer. Amplification and HRM analysis were carried out on a Gentier 48E real-time PCR system (Xi’an Tianlong Science and Technology Co., Ltd., Xi’an, China) under the following conditions: 95 °C for 5 min, followed by 20 cycles of outer preamplification (95 °C for 15 s and 72 °C for 30 s, without fluorescence acquisition), 40 cycles of nested real-time amplification (95 °C for 15 s, 57 °C for 20 s, and 72 °C for 30 s, with fluorescence acquisition at 72 °C), and HRM analysis from 55 °C to 98 °C with 0.5 °C increments. A sample was interpreted as positive when Ct was <33 and the characteristic HRM profile was obtained. The Ct <33 threshold was selected as a conservative operational cut-off based on assay optimization, in which late signals above this range were less reproducible and more difficult to distinguish from weak non-specific amplification. The assay was used in this study as an in-house molecular screening tool for paired workflow comparison. Key analytical characteristics of the assay, including analytical sensitivity, matrix blank behavior, carryover assessment, analytical specificity, template-input optimization, and clinical agreement, are summarized in Supplementary Table S1.

### 2.6. PCR Detection of Pentatrichomonas hominis

Detection of *Pentatrichomonas hominis* was performed using a species-specific PCR assay targeting the 18S rRNA gene, based on the feline fecal assay described by Gookin et al. [17] and the optimization work reported for the same target/primer system [18]. The primer pair used was Th3 (5′-TGTAAACGATGCCGACAGAG-3′) and Th5 (5′-CAACACTGAAGCCAATGCGAGC-3′), generating a 339 bp product. PCR cycling conditions reported for this assay consisted of initial denaturation followed by 45 repeated cycles including denaturation at 95 °C for 30 s, annealing at 64 °C for 1 min., and extension at 72 °C for 90 s, yielding the 339 bp diagnostic amplicon.

### 2.7. PCR Detection of Cryptosporidium *spp*.

Detection of *Cryptosporidium* spp. was performed using the one-tube nested real-time PCR-HRM assay described by Santana et al. [19]. This assay targets the 18S rRNA gene and uses the outer primer pair NRT18SF (5′-GTTGTTGCAGTTAAAAAGCTCGTAGTTGGATT-3′) and NRT18SR (5′-ACTTTGATTTCTCATAAGGTGCTGAAGGAGT-3′), together with the inner primer pair CPB-DIAGF (5′-AAGCTCGTAGTTGGATTTCTG-3′) and CPB-DIAGR (5′-TAAGGTGCTGAAGGAGTAAGG-3′) [20]. The assay amplifies a fragment of approximately 428–457 bp within the 18S rRNA locus. The same DNA extract was used for this assay as for the other PCR tests included in the study. Samples were considered positive when Ct was <33. Positive amplicons were subsequently subjected to sequencing of the 18S rRNA fragment for confirmation.

### 2.8. PCR Detection and Genotyping of Giardia duodenalis

Genotyping of *Giardia duodenalis* was based on amplification of the beta-giardin locus using the nested PCR approach described by Lalle et al. [21]. In the primary PCR, primers G7 (5′-AAGCCCGACGACCTCACCCGCAGTGC-3′) and G759 (5′-GAGGCCGCCCTGGATCTTCGAGACGAC-3′) were used. In the secondary PCR, primers 511F (5′-GAACGAACGAGATCGAGGTCCG-3′) and 511R (5′-CTCGACGAGCTTCGTGTT-3′) were used, generating an amplicon of approximately 511 bp. Positive beta-giardin amplicons were subjected to Sanger sequencing for genotype confirmation.

### 2.9. Antigen Testing and Microscopy

Routine fecal examination included antigen-based testing for *Giardia duodenalis* and *Cryptosporidium* spp. using the Stick Crypto-*Giardia* Operon immunochromatographic assay (Operon, Zaragoza, Spain). In addition, fecal samples were examined for the presence of *G. duodenalis* cysts using a zinc sulfate flotation solution with a specific gravity of 1.31 g/cm^3^.

### 2.10. Interpretation of PCR Results

For all real-time PCR-HRM assays included in the study, a sample was interpreted as positive only when amplification occurred before Ct 33 and was accompanied by the expected assay-specific HRM profile. Thus, Ct alone was not used as the sole criterion for positivity. The Ct <33 threshold was selected as a conservative operational cut-off to reduce the risk of interpreting late, weak, or non-specific amplification as positive. For *Cryptosporidium* spp., positive amplicons were subsequently confirmed by Sanger sequencing of the 18S rRNA fragment. For *Giardia duodenalis*, beta-giardin nested PCR amplicons of the expected size were sequenced for assemblage confirmation. For conventional and nested PCR assays, amplification products were visualized by 1.5% agarose gel electrophoresis under UV light. Each fecal sample was extracted once per workflow after homogenization and paired aliquoting. Weak or borderline amplification results were repeated when sufficient DNA was available and were considered positive only if the predefined molecular criteria were met.

### 2.11. Sequencing and Phylogenetic Analysis

Positive *Giardia* beta-giardin amplicons and *Cryptosporidium* 18S rRNA amplicons were subjected to Sanger dideoxy sequencing to determine assemblages and species, respectively. The obtained sequences were edited and compared with homologous reference sequences deposited in the GenBank database using the Basic Local Alignment Search Tool (BLAST). To further assess their phylogenetic relationships, neighbor-joining trees were constructed in MEGA6 using the Kimura two-parameter substitution model [22,23]. Tree robustness was assessed by bootstrap analysis with 500 replicates.

### 2.12. Post-Extraction Spike-Recovery Experiment

To directly evaluate post-extraction PCR inhibition, a spike-recovery experiment was performed on selected paired DNA extracts. A standardized amount of *Cryptosporidium meleagridis* 18S qPCR-positive control DNA was added to reactions containing 2 µL of DNA extract obtained either by direct extraction or by cleanup-based extraction. The same spike working dilution was used within each run. Spike reactions prepared in nuclease-free water were included in each run and served as run-specific no-inhibition references. No-template controls and spike reactions containing a *Cryptosporidium*-negative fecal DNA extract were also included as control reactions. The experiment included 44 paired extracts tested across five runs. Because the Ct value of the spike + water control showed a gradual run-to-run increase, all inhibition estimates were normalized to the mean spike + water Ct obtained within the same run. PCR inhibition was expressed as ΔCt, calculated as the Ct in the presence of fecal DNA extract minus the mean Ct of the spike + water control from the same run. Reactions without amplification by the end of the 35-cycle fluorescence acquisition stage were conservatively coded as Ct = 35.0. Spike recovery was estimated as 2^−ΔCt^ × 100%, with values capped at 100%. Extracts with ΔCt > 1 and ΔCt > 3 were classified operationally as showing relevant and strong inhibition, respectively.

### 2.13. Statistical Analysis

Continuous variables were assessed for distribution and are presented as median and interquartile range (IQR), because most laboratory parameters showed a non-normal distribution. Paired comparisons between direct extraction and extraction preceded by preliminary fecal cleanup were performed using the Wilcoxon signed-rank test. Differences in paired binary outcomes, including PCR positivity obtained before and after cleanup and the frequency of extracts classified as strongly inhibited in the spike-recovery experiment, were evaluated using the exact McNemar test. Comparisons between independent groups were performed using the Mann–Whitney U test for two-group analyses and the Kruskal–Wallis test for analyses involving more than two groups. Associations between categorical variables were assessed using Fisher’s exact test or the chi-square test, as appropriate.

For the spike-recovery experiment, direct and cleanup ΔCt values and calculated spike-recovery percentages were compared using paired Wilcoxon signed-rank tests. Correlations between A260/230 ratios and ΔCt inhibition values were assessed using Spearman’s rank correlation coefficient. Apparent sensitivity, apparent specificity, positive predictive value (PPV), and negative predictive value (NPV) of antigen assays and microscopy were calculated relative to selected comparators and are reported with 95% exact binomial confidence intervals. Because no independent diagnostic gold standard was available, these estimates were interpreted as apparent performance and agreement relative to the selected comparator rather than as definitive diagnostic accuracy. Cohen’s kappa coefficient was calculated to describe agreement. No formal statistical comparison between kappa coefficients was performed. Secondary analyses involving stool characteristics and host variables were considered exploratory and interpreted cautiously. All tests were two-sided, and *p*-values < 0.05 were considered statistically significant. Statistical analyses were performed using JASP, version 0.19.3. No formal adjustment for multiple comparisons was applied. Therefore, secondary analyses involving age, sex, stool consistency, and stool color were interpreted as exploratory.

## 3. Results

### 3.1. Study Population

A total of 105 feline fecal samples were included in the study. The study population comprised 60 males (57.1%) and 45 females (42.9%). Age ranged from 0.17 to 16.83 years, with a median age of 2.0 years (IQR: 1.0–6.0 years) and a mean age of 3.95 years. Twenty-five cats were younger than 1 year, 40 were aged 1–3 years, 19 were aged >3–7 years, and 21 were older than 7 years (Table 1). Regarding stool consistency, 19 samples were classified as loose, 34 as pasty, 24 as firm, 17 as compact, 5 as hard firm, 2 as soft compact, and 4 as fatty. Stool color was described as brown in 36 samples, light brown in 33, very dark in 29, and gray in 7 samples.

### 3.2. Effect of Preliminary Cleanup on DNA Yield and Purity

Preliminary cleanup significantly improved both DNA extraction and DNA purity. Median DNA concentration increased from 61.16 ng/µL after direct extraction to 164.26 ng/µL after cleanup-based extraction (Wilcoxon signed-rank test, *p* = 2.05 × 10^−11^). Likewise, the median A260/280 ratio increased from 1.80 to 2.06 (*p* = 3.62 × 10^−15^), and the median A260/230 ratio increased from 1.24 to 1.81 (*p* = 8.06 × 10^−13^). These findings indicate that the chloroform:methanol cleanup step improved not only the amount of recovered DNA but also the spectrophotometric quality of the extracts.

### 3.3. PCR Detection of Enteric Protozoa Before and After Cleanup

**Figure 2:**
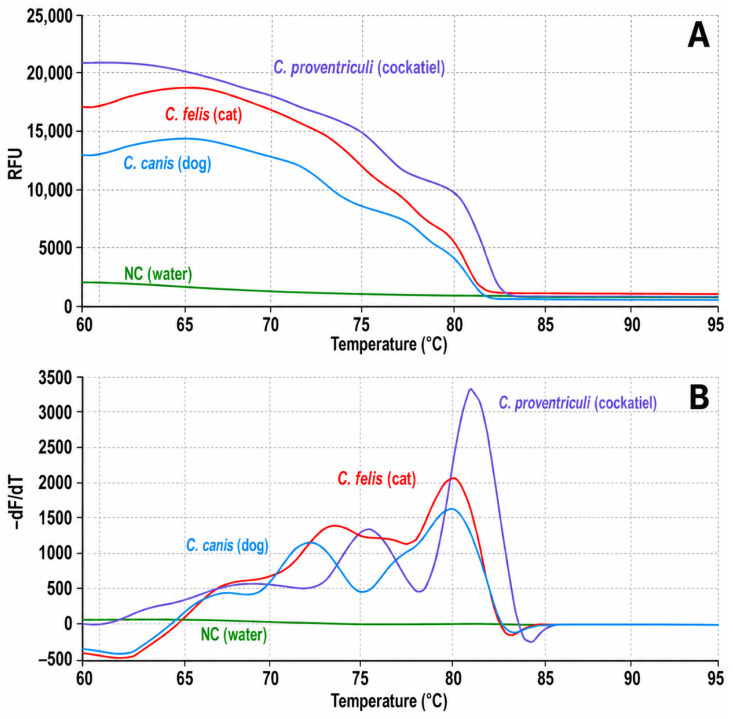
High-resolution melting (HRM) profiles obtained for representative *Cryptosporidium* amplicons. (**A**) Normalized melting curves showing fluorescence loss (RFU) during gradual temperature increase for *Cryptosporidium felis* from feline feces, *Cryptosporidium canis* from canine feces, *Cryptosporidium proventriculi* from a cockatiel sample, and the negative control. (**B**) Corresponding derivative melting peak plots (−dF/dT) generated from the same reactions. The analyzed *Cryptosporidium* species showed distinct HRM patterns, supporting species-level discrimination among the tested controls. The negative control remained flat and did not generate a specific melting peak. HRM results were interpreted together with amplification curves and sequencing confirmation of positive feline samples.

**Figure 3:**
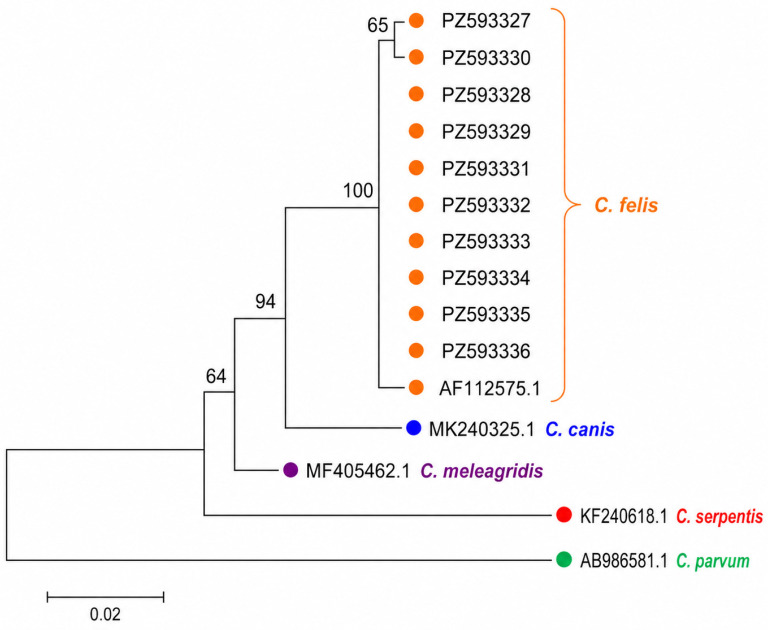
Neighbor-joining phylogenetic tree based on sequences obtained from feline fecal samples positive for *Cryptosporidium* spp. Phylogenetic analysis of *Cryptosporidium* spp. based on the partial 18S rRNA gene. All sequences generated in this study clustered with the *Cryptosporidium felis* reference sequence, confirming species assignment of the feline isolates to *C. felis*. The sequences were deposited in GenBank under accession numbers PZ593327-PZ593336. The tree was constructed in MEGA6 using the neighbor-joining method with the Kimura two-parameter model. Bootstrap support values based on 500 replicates are shown next to the branches. Scale bars indicate the number of nucleotide substitutions per site.

For *Cryptosporidium* spp., 7/105 samples (6.7%) were PCR-positive after direct extraction, compared with 10/105 (9.5%) after extraction preceded by cleanup (Table 2, Figure 2). In paired analysis, seven samples were positive in both workflows, three were positive only after cleanup, and none were positive only after direct extraction. The difference in positivity rate did not reach statistical significance in the exact McNemar test (*p* = 0.2500). However, among samples positive in both workflows, cleanup significantly reduced Ct values. Median Ct decreased from 22.215 to 18.574, with a median paired Ct improvement of 2.523 cycles (Wilcoxon signed-rank test, *p* = 0.0156) (Table 3). All 10 *Cryptosporidium*-positive amplicons were confirmed by sequencing as *Cryptosporidium felis*. The partial 18S rRNA sequences generated in this study were deposited in GenBank under accession numbers PZ593327-PZ593336 (Figure 3).

For *Tritrichomonas foetus*, 3/105 samples (2.9%) were positive both before and after cleanup (Table 2). No sample converted from negative to positive or from positive to negative, and the exact McNemar test was not significant (*p* = 1.0000). Ct values were lower after cleanup in the paired positive samples, with a median decrease from 27.119 to 17.588 and a median paired Ct improvement of 9.531 cycles. However, because only three paired positive samples were available, this analysis should be regarded as descriptive and statistically underpowered rather than as robust evidence of a target-specific effect.

No *Pentatrichomonas hominis*-positive samples were detected in either extraction workflow.

**Figure 4:**
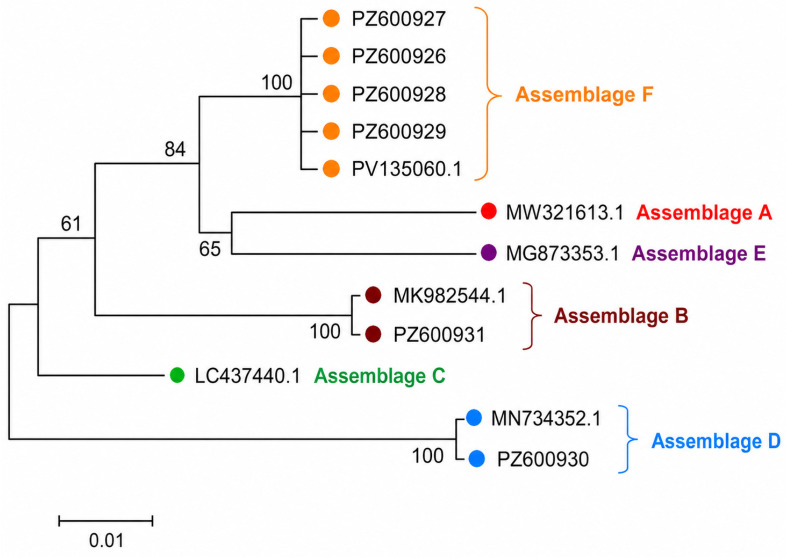
Neighbor-joining phylogenetic tree based on sequences obtained from feline fecal samples positive for *Giardia duodenalis*. Phylogenetic analysis was performed using partial beta-giardin gene sequences. Four sequences generated in this study clustered with assemblage F (PZ600926-PZ600929), one sequence clustered with assemblage B (PZ600931), and one sequence clustered with assemblage D (PZ600930). The sequences were deposited in GenBank under accession numbers PZ600926-PZ600931. The tree was constructed in MEGA6 using the neighbor-joining method with the Kimura two-parameter model. Bootstrap support values based on 500 replicates are shown next to the branches. Scale bars indicate the number of nucleotide substitutions per site.

For *Giardia duodenalis*, beta-giardin PCR positivity increased from 1/105 (1.0%) after direct extraction to 6/105 (5.7%) after cleanup-based extraction. In paired analysis, one sample was positive in both workflows, five additional samples were positive only after cleanup, and no sample was positive only after direct extraction (Table 2). This difference did not reach conventional statistical significance (exact McNemar *p* = 0.0625), and the result should therefore be interpreted cautiously as preliminary evidence of improved genotyping success after cleanup. Sanger sequencing of the six beta-giardin amplicons showed mixed assemblage assignment. Four sequences clustered with *G. duodenalis* assemblage F (PZ600926-PZ600929), one sequence clustered with assemblage B (PZ600931), and one sequence clustered with assemblage D (PZ600930). The sequences obtained in this study were deposited in GenBank under accession numbers PZ600926-PZ600931 (Figure 4).

### 3.4. Stool Consistency, Stool Color, and Molecular Detection

For statistical analysis, stool consistency was grouped into unformed stools (loose + pasty) and formed stools (firm + compact + hard firm + soft compact), whereas fatty samples were assessed descriptively because of their low number. No significant association was found between stool consistency and PCR positivity for *Cryptosporidium* spp. (13.2% in unformed vs. 6.3% in formed stools; Fisher’s exact test, *p* = 0.325), *T. foetus* (5.7% vs. 0.0%; *p* = 0.244), or *Giardia* beta-giardin PCR (7.5% vs. 4.2%; *p* = 0.680). Similarly, stool consistency was not significantly associated with DNA concentration, A260/280, or A260/230 values, either before or after cleanup (all *p* > 0.05).

In contrast, stool color was significantly associated with baseline DNA extraction performance before cleanup. DNA concentration differed significantly across stool color groups (Kruskal–Wallis test, *p* = 0.00051), as did A260/280 (*p* = 0.00031) and A260/230 (*p* = 0.00007). Very dark samples showed the poorest baseline extraction parameters. After cleanup, the effect of stool color became much weaker: DNA concentration still differed between color groups (*p* = 0.0137), whereas A260/280 was no longer clearly significant (*p* = 0.0535) and A260/230 showed no significant difference (*p* = 0.451). Stool color was not significantly associated with PCR positivity for *Cryptosporidium* spp., *T. foetus*, or *Giardia*.

Overall, these findings suggest that stool color may reflect matrix-related factors affecting DNA extraction quality, whereas stool consistency alone is a relatively weak predictor of downstream molecular detectability.

### 3.5. Age and Sex in Relation to PCR Positivity

In exploratory unadjusted analyses, cats positive for *Cryptosporidium* spp. were younger than PCR-negative cats. The median age of PCR-positive cats was 0.96 years, compared with 2.75 years in PCR-negative animals (Mann–Whitney U test, *p* = 0.0064). A similar descriptive age-related pattern was observed for *Tritrichomonas foetus*, with a median age of 0.75 years in PCR-positive cats and 2.21 years in PCR-negative cats. However, because only three cats were positive for *T. foetus*, this finding should be interpreted with particular caution despite the nominal *p* value of 0.033, and should be regarded as hypothesis-generating rather than confirmatory. For *Giardia duodenalis* beta-giardin PCR, age did not differ significantly between PCR-positive and PCR-negative cats (median 1.25 vs. 2.42 years; *p* = 0.208).

No statistically significant associations were observed between sex and PCR positivity for *Cryptosporidium* spp. (*p* = 0.0947), *T. foetus* (*p* = 0.258), or *G. duodenalis* beta-giardin PCR (*p* = 0.398). These host-factor analyses were secondary to the main paired workflow comparison and should therefore be interpreted as exploratory.

### 3.6. Agreement of Antigen Assays and Microscopy with PCR

For *Giardia*, the antigen test showed full sensitivity relative to microscopy (100%) and high specificity (96.9%), with a positive predictive value (PPV) of 75.0%, a negative predictive value (NPV) of 100%, and Cohen’s kappa of 0.842. When compared with cleanup-based beta-giardin PCR, the *Giardia* antigen test also showed 100% sensitivity and 93.9% specificity, but PPV decreased to 50.0%, while NPV remained 100% (kappa = 0.639). Microscopy showed 100% sensitivity and 97.0% specificity relative to cleanup-based beta-giardin PCR, with PPV of 66.7%, NPV of 100%, and kappa of 0.785.

For *Cryptosporidium*, the antigen test showed 100% sensitivity and 95.8% specificity when compared with cleanup-based PCR, with a PPV of 71.4%, NPV of 100%, and kappa of 0.813. When compared with direct-extraction PCR, the *Cryptosporidium* antigen assay also showed 100% sensitivity, but specificity decreased to 92.9%, PPV to 50.0%, and kappa to 0.634 (Table 4).

Because no independent diagnostic gold standard was available, these estimates should be interpreted as apparent performance and agreement relative to the selected comparator rather than as definitive diagnostic accuracy. Agreement estimates were interpreted descriptively. No formal statistical comparison between kappa coefficients was performed.

### 3.7. Post-Extraction Spike-Recovery Analysis

Fecal DNA extracts were tested for their ability to directly inhibit PCR amplification using a post-extraction spike-recovery experiment. The analysis included 44 paired extracts tested across five runs. The mean Ct of the spike + water control increased gradually across runs, from 26.955 in Run 1 to 28.836 in Run 5; therefore, all ΔCt values were calculated relative to the run-specific spike + water mean Ct.

Direct-extraction DNA markedly delayed amplification of the exogenous *Cryptosporidium* 18S spike. The median Ct was 34.51 for direct extracts and 30.72 for cleanup-based extracts. Median ΔCt inhibition was 6.37 cycles for direct extracts and 2.75 cycles for cleanup-based extracts. This reduction in Ct delay after cleanup was statistically significant in paired analysis (Wilcoxon signed-rank test, *p* = 1.14 × 10^−13^). Consistently, the estimated median spike recovery increased from 1.21% in direct extracts to 14.90% after cleanup.

Relevant inhibition, defined operationally as ΔCt > 1, was observed in 44/44 direct extracts and in 37/44 cleanup-based extracts. Strong inhibition, defined as ΔCt > 3, was observed in 43/44 direct extracts and in 20/44 cleanup-based extracts (exact McNemar test, *p* = 2.38 × 10^−7^). Direct-extraction A260/230 values were moderately and inversely correlated with ΔCt inhibition (Spearman ρ = −0.495, *p* = 0.000639), whereas the corresponding correlation after cleanup was weaker and not statistically significant (ρ = −0.179, *p* = 0.245). These results indicate that direct feline fecal DNA extracts exhibited substantial post-extraction PCR-inhibitory activity and that the cleanup workflow significantly diminished this inhibitory effect in the *Cryptosporidium* 18S PCR system tested (Tables S2–S5).

## 4. Discussion

The main finding of this paired methodological study is that preliminary chloroform:methanol cleanup improved the analytical performance of feline fecal DNA extraction and downstream molecular amplifiability. This effect was supported by three complementary layers of evidence. First, cleanup significantly improved spectrophotometric extract quality, including DNA concentration, A260/280, and A260/230 ratios. Second, cleanup was associated with additional molecular detections of *Cryptosporidium* spp. and *Giardia duodenalis* and significantly lower Ct values for *Cryptosporidium* spp. among paired positive samples. Third, and most importantly in relation to PCR inhibition, the post-extraction spike-recovery experiment directly demonstrated that direct feline fecal DNA extracts delayed amplification of a fixed exogenous *Cryptosporidium* 18S spike, whereas cleanup-based extracts showed significantly lower Ct delay and higher calculated spike recovery. Together, these findings indicate that the fecal matrix represented a major analytical bottleneck in this workflow and that the cleanup step reduced this matrix-associated effect.

The spike-recovery experiment provides direct evidence that post-extraction PCR inhibition was present in the tested feline fecal DNA extracts. Direct extracts showed a median ΔCt delay of 6.37 cycles relative to the run-specific spike + water control, whereas cleanup-based extracts showed a significantly lower median ΔCt of 2.75 cycles. Estimated median spike recovery increased from 1.21% in direct extracts to 14.90% after cleanup, and the frequency of strongly inhibited extracts, defined operationally as ΔCt > 3, decreased from 43/44 direct extracts to 20/44 cleanup-based extracts. This is consistent with the well-established observation that fecal PCR may be impaired by bile salts, bilirubin, complex polysaccharides, lipids, hemoglobin degradation products, plant- or diet-derived compounds, and other organic substances that can affect DNA polymerase activity, nucleic acid integrity, fluorescence chemistry, or primer annealing [2,3]. The inverse correlation observed between direct-extraction A260/230 values and ΔCt inhibition further supports the biological plausibility of the finding, because low A260/230 ratios commonly reflect carryover of salts, organic compounds, and other non-protein contaminants that may interfere with PCR [2,3].

The relevance of the pre-analytical phase in fecal PCR workflows is further supported by studies performed in other host species and diagnostic contexts. In human stool, PCR inhibition has long been recognized as a major analytical problem, and sample-preparation procedures designed to remove inhibitory compounds were shown to improve amplification from inhibitor-rich fecal homogenates [24]. In parasitological diagnostics, pre-PCR processing of stool has also been shown to affect the molecular detection of *Giardia intestinalis*/*Giardia duodenalis*, with selected concentration techniques influencing PCR performance and highlighting that fecal preparation is not analytically neutral [4]. Similarly, protocols developed for DNA extraction from protozoan cysts and oocysts in feces emphasize that molecular detection may be limited both by incomplete disruption of resistant parasite stages and by residual inhibitors carried into the amplification reaction [25]. For *Cryptosporidium*, comparative work on mechanical stool pretreatment demonstrated that DNA recovery from oocysts depends strongly on the disruption protocol and bead matrix used before extraction [26]. This is consistent with feline molecular diagnostics, where different commercial DNA extraction methods were previously shown to affect PCR detection of *Tritrichomonas foetus* in feline stool specimens [27]. Collectively, these studies indicate that fecal PCR results are strongly influenced by sample preparation, inhibitor reduction, target release, and extraction chemistry. The present study extends this concept to feline fecal protozoal diagnostics by showing, in paired samples, that chloroform:methanol cleanup improved DNA extract quality and significantly reduced post-extraction PCR inhibition, as demonstrated by the spike-recovery experiment. However, because fecal pretreatment can also influence target DNA recovery or loss, the observed improvement should be interpreted as a workflow-level effect resulting from reduced matrix interference and improved amplifiability rather than as evidence of a single isolated mechanism.

At the same time, the mechanism of improvement should be interpreted with appropriate caution. The paired comparison used the same fecal mass and the same template volume in PCR, but cleanup also increased DNA yield. Therefore, lower Ct values in parasite-positive samples may reflect a combination of reduced inhibition, improved recovery of amplifiable DNA, improved removal of organic contaminants, and potentially more efficient release or retention of target DNA. The spike-recovery experiment specifically assessed post-extraction PCR inhibition by adding a fixed exogenous target to already extracted DNA. It does not fully measure extraction efficiency, lysis efficiency, or possible target DNA loss during cleanup. Organic solvent treatment may theoretically reduce recovery of some free extracellular DNA or low-abundance target DNA, even though no direct-extraction-only positive sample was observed in the present dataset. Therefore, the improvement observed here should be interpreted as a workflow-level effect rather than as proof of a single isolated mechanism.

For *Cryptosporidium* spp., cleanup increased PCR positivity from 7/105 to 10/105 samples and significantly lowered Ct values among samples positive in both workflows. Although the increase in positivity rate did not reach statistical significance, the combination of additional detections, lower Ct values, sequence confirmation, and spike-recovery data supports the interpretation that cleanup improved the molecular suitability of feline fecal extracts. All 10 *Cryptosporidium*-positive amplicons were confirmed by sequencing as *Cryptosporidium felis* and deposited in GenBank under accession numbers PZ593327-PZ593336. The one-tube nested real-time PCR-HRM assay originally described for avian fecal samples was successfully applied to feline fecal DNA extracts in the present study [19]. However, these findings should not be interpreted as full feline-specific validation across all *Cryptosporidium* species potentially detected in cats, such as *C. parvum*, *C. canis*, or other less frequent species. The HRM profiles supported discrimination among the tested controls, but broader validation using a larger panel of *Cryptosporidium* species and feline fecal matrices would be needed to define the full analytical specificity and discriminatory capacity of this assay in cats.

The *Giardia duodenalis* findings are relevant mainly because they concern genotyping success. Cleanup increased beta-giardin PCR positivity from 1/105 to 6/105 samples. This difference did not reach conventional statistical significance and should therefore be interpreted cautiously as preliminary evidence of improved genotyping success after cleanup. Sequencing showed that four beta-giardin amplicons clustered with assemblage F (PZ600926-PZ600929), one with assemblage B (PZ600931), and one with assemblage D (PZ600930). Assemblage F is generally regarded as the cat-adapted assemblage, whereas assemblage B is more frequently discussed in the context of zoonotic transmission. The detection of assemblage D, which is usually associated with canids, is epidemiologically noteworthy in a feline fecal sample. However, because only a single assemblage D sequence was identified and only one locus was analyzed, this finding should not be overinterpreted. Possible explanations include true infection after cross-host exposure, transient passage of cysts, environmental contamination, or undetected mixed infection. Multilocus genotyping and a larger number of positive feline samples would be required to clarify the epidemiological relevance of this observation [9,10,11]. These results also support the value of molecular typing, because microscopy and coproantigen testing cannot determine assemblage identity.

For *Tritrichomonas foetus*, the number of positive samples was low, which limits statistical interpretation. The same three samples were positive before and after cleanup, and no change in positivity rate was observed. Ct values were lower after cleanup in these paired positive samples, but because only three positive pairs were available, this result should be treated as descriptive rather than statistically robust. Nevertheless, the direction of the Ct shift is consistent with the broader spike-recovery results and supports the general interpretation that cleanup can improve amplifiability in inhibitor-rich feline fecal extracts. The inclusion of *Pentatrichomonas hominis* in the panel was also justified diagnostically. Non-*T. foetus* trichomonads, including *P. hominis*, may occur in feline fecal samples, and direct examination or fecal culture may not reliably distinguish *P. hominis* from *T. foetus* without molecular confirmation [17,28]. PCR-based assays are therefore important for differentiating trichomonad species in feline fecal diagnostics, particularly when motile trophozoites or trichomonad-like organisms are observed [17,28]. No *P. hominis*-positive samples were detected in either workflow. This result should not be interpreted as evidence of absence from the broader feline population, because the study was not designed as a prevalence survey for this organism, and no independent analytical sensitivity comparison was performed for this target.

Stool consistency alone was not significantly associated with DNA yield, extract purity, or parasite detection. This suggests that macroscopic consistency is a relatively crude indicator of the molecular suitability of feline fecal material. Fecal consistency is widely used as a practical clinical descriptor of gastrointestinal function in cats, and standardized fecal scoring systems have been applied in feline studies to classify stool form and monitor gastrointestinal status [29,30]. However, consistency mainly reflects water content and stool form and does not necessarily capture the biochemical composition of the sample. Two fecal samples with similar consistency may differ substantially in bile acid content, lipid burden, host-derived material, dietary residues, microbial biomass, and other matrix components relevant to DNA extraction and PCR inhibition.

In contrast, stool color was associated with baseline extraction parameters before cleanup, particularly in very dark samples, which showed poorer DNA yield and purity ratios. This observation is biologically plausible because feline feces can vary in lipid-, sterol-, and bile acid-associated composition, and metabolomic studies in cats with chronic enteropathy have demonstrated altered fecal concentrations of long-chain fatty acids, sterols, and unconjugated bile acids [5,6]. Such compounds may contribute to matrix complexity and may indirectly influence DNA extraction quality or PCR amplifiability. However, the interpretation of stool color in the present study must remain cautious. Color was assessed macroscopically and was not standardized using a color chart or digital colorimetry. Moreover, bile acids, bilirubin, hemoglobin derivatives, sterols, lipids, and polysaccharides were not chemically quantified. Therefore, stool color cannot be considered a validated marker of inhibitor burden. It should be interpreted only as an exploratory laboratory observation suggesting that some visually unfavorable fecal matrices may be more difficult for DNA extraction and that cleanup may reduce part of this matrix-related effect.

The age-related findings were biologically coherent. Cats positive for *Cryptosporidium* spp. and *T. foetus* were significantly younger than PCR-negative cats. Young age and high-density environments are recognized risk factors for feline trichomonosis and have repeatedly been associated with enteric protozoal infections in cats, particularly in animals from multicat households, shelters, catteries, or breeding settings [7,8]. For *Cryptosporidium*, systematic and review-based evidence also indicates that prevalence varies by host and age group and that younger cats may contribute disproportionately to the burden of infection [9,14]. The clustering of positive PCR results in younger animals supports the biological credibility of the dataset and argues against the additional detections after cleanup being merely stochastic analytical events. However, because the study was designed as a methodological workflow comparison rather than an epidemiological survey, age-related results should be interpreted as supportive rather than as primary epidemiological findings.

The comparison between immunoassays, microscopy, and PCR should also be interpreted cautiously. In this study, PCR after cleanup was used as a molecular comparator, not as an independent diagnostic gold standard. Therefore, the calculated sensitivity, specificity, PPV, and NPVs represent apparent performance relative to the selected comparator rather than definitive diagnostic accuracy. The numerically different kappa values observed across comparisons may reflect changes in the analytical quality of the molecular comparator, but no formal statistical comparison between kappa coefficients was performed. More generally, microscopy, coproantigen assays, and PCR detect different biological signals. Microscopy detects visible cysts or oocysts, antigen tests detect parasite-derived proteins, and PCR detects nucleic acids. These methods should therefore be regarded as complementary rather than strictly interchangeable diagnostic approaches [12,13]. This is particularly relevant in fecal parasitology, where intermittent shedding, low parasite burden, antigen persistence, DNA degradation, and PCR inhibition may all contribute to discordant results.

From a routine laboratory perspective, the present findings are encouraging because the tested cleanup step improved molecular workflow performance without changing the downstream assay design. In commercial veterinary diagnostics, pre-analytical optimization may be easier to implement than changing amplification chemistry, introducing multiple rescue dilutions, or replacing validated diagnostic algorithms. The use of chloroform:methanol cleanup may be particularly relevant for fecal samples with low A260/230 values or visually unfavorable matrices. However, the procedure adds handling time, requires organic solvent use, and should be performed with appropriate laboratory safety measures. Its practical implementation should therefore be balanced against sample throughput, laboratory infrastructure, occupational safety, and the clinical importance of avoiding false-negative molecular results.

This study has several limitations. First, the number of positive *Tritrichomonas foetus* and *Giardia duodenalis* beta-giardin samples was low, which limited the statistical power of target-specific comparisons. The *T. foetus* Ct analysis should therefore be considered descriptive, and the increase in *Giardia* beta-giardin PCR positivity should be interpreted as preliminary. Second, although the spike-recovery experiment directly demonstrated post-extraction PCR inhibition and its reduction after cleanup, it assessed inhibition in already extracted DNA and did not measure total extraction efficiency, lysis efficiency, or target DNA loss during cleanup. Third, the same template volume was used for PCR in both workflows, while cleanup increased DNA yield; therefore, lower Ct values in parasite-positive samples may partly reflect increased recovery of amplifiable target DNA in addition to reduced inhibition. Fourth, stool color was assessed macroscopically and was not validated using standardized color charts, digital colorimetry, or biochemical measurement of inhibitors such as bile acids, bilirubin, hemoglobin derivatives, sterols, lipids, or polysaccharides. Fifth, only one cleanup protocol and one automated extraction platform were evaluated, so the findings should not be automatically generalized to all fecal DNA extraction systems, commercial kits, or manual protocols. Finally, the *Cryptosporidium* HRM assay was successfully applied to feline fecal samples and confirmed by sequencing, but broader feline-specific validation involving multiple *Cryptosporidium* species would be required to fully define assay specificity and discriminatory performance in cats.

Overall, the results support chloroform:methanol cleanup as a promising pre-analytical strategy for improving feline fecal molecular diagnostics. The strongest evidence is the reduction in post-extraction PCR inhibition demonstrated by spike-recovery, together with improved spectrophotometric extract quality and improved molecular detectability in selected protozoan assays. The findings should be interpreted as workflow-level evidence rather than as universal validation of the method. Further studies using larger positive sample sets, input-normalized PCR, broader inhibition controls, additional extraction platforms, and standardized biochemical characterization of fecal matrices are warranted.

## 5. Conclusions

This paired methodological study shows that chloroform:methanol pre-analytical cleanup improves feline fecal DNA extract quality and reduces post-extraction PCR inhibition in the tested workflow. Cleanup increased DNA concentration and improved A260/280 and A260/230 ratios, was associated with additional *Cryptosporidium* and *Giardia* detections, significantly lowered *Cryptosporidium* Ct values among paired positives, and markedly improved spike recovery in a direct inhibition experiment. All *Cryptosporidium*-positive samples were confirmed as *Cryptosporidium felis*. Among the six *Giardia* beta-giardin sequences, four belonged to assemblage F, whereas one sequence each clustered with assemblages B and D. These findings support cleanup as a promising pre-analytical strategy for improving molecular analysis of feline fecal samples. However, the results should be interpreted as workflow-level evidence rather than as universal validation of the method. Further studies using larger positive sample sets, input-normalized PCR, broader inhibition controls, multiple extraction platforms, and additional *Cryptosporidium* and *Giardia* targets are needed before routine implementation can be broadly recommended.

## Figures and Tables

**Table 1:** Characteristics of the study population.

Characteristic	Value
Total cats	105
Male	60 (57.1%)
Female	45 (42.9%)
Age, years, mean ± SD	3.95 ± 3.79
Age, years, median (IQR)	2.00 (1.00–6.00)
Age range, years	0.17–16.83
Age < 1 year	25 (23.8%)
Age 1–3 years	40 (38.1%)
Age > 3–7 years	19 (18.1%)
Age > 7 years	21 (20.0%)

**Table 2:** Paired comparison of molecular detection before and after preliminary cleanup.

Target	Direct Extraction Positive, *n* (%)	Cleanup Extraction Positive, *n* (%)	Positive in Both Workflows	Positive Only After Cleanup	Positive Only After Direct Extraction	Exact McNemar *p* Value
*Cryptosporidium* spp.	7/105 (6.7%)	10/105 (9.5%)	7	3	0	0.2500
*T. foetus*	3/105 (2.9%)	3/105 (2.9%)	3	0	0	1.0000
*P. hominis*	0/105 (0.0%)	0/105 (0.0%)	0	0	0	1.0000
*G. duodenalis*	1/105 (1.0%)	6/105 (5.7%)	1	5	0	0.0625

**Table 3:** Ct comparison in paired PCR-positive samples.

Target	n Paired Positives	Median Ct, Direct Extraction	Median Ct, Cleanup-Based Extraction	Median Paired Ct Difference	*p* Value
*Cryptosporidium* spp.	7	22.215	18.574	2.523	0.0156
*T. foetus*	3	27.119	17.588	9.531	0.2500

**Table 4:** Performance of antigen tests and microscopy relative to molecular assays.

Comparison	Sensitivity	Specificity	PPV	NPV	Cohen’s Kappa
*Giardia* Ag vs. microscopy	100.0% (66.4–100.0)	96.9% (91.1–99.4)	75.0% (42.8–94.5)	100.0% (96.1–100.0)	0.842
*Giardia* Ag vs. PCR after cleanup	100.0% (54.1–100.0)	93.9% (87.3–97.7)	50.0% (21.1–78.9)	100.0% (96.1–100.0)	0.639
*Giardia* microscopy vs. PCR after cleanup	100.0% (54.1–100.0)	97.0% (91.4–99.4)	66.7% (29.9–92.5)	100.0% (96.2–100.0)	0.785
*Cryptosporidium* Ag vs. PCR after cleanup	100.0% (69.2–100.0)	95.8% (89.6–98.8)	71.4% (41.9–91.6)	100.0% (96.0–100.0)	0.813
*Cryptosporidium* Ag vs. direct-extraction PCR	100.0% (59.0–100.0)	92.9% (85.8–97.1)	50.0% (23.0–77.0)	100.0% (96.0–100.0)	0.634

Note: Values are percentages with 95% exact binomial confidence intervals in parentheses.

## Data Availability

The sequences generated in this study were deposited in GenBank. *Cryptosporidium felis* partial 18S rRNA sequences are available under accession numbers PZ593327-PZ593336. *Giardia duodenalis* beta-giardin sequences are available under accession numbers PZ600926-PZ600931, including four assemblage F sequences (PZ600926-PZ600929), one assemblage B sequence (PZ600931), and one assemblage D sequence (PZ600930). The spike-recovery dataset supporting the analysis of post-extraction PCR inhibition is provided in the Supplementary Materials.

## Supplementary Materials

The following supporting information can be downloaded at: https://www.mdpi.com/article/10.3390/cimb48070707/s1.

## Abbreviations

The following abbreviations are used in this manuscript:

bp	base pairs
Ct	cycle threshold
DNA	deoxyribonucleic acid
HRM	high-resolution melting
IQR	interquartile range
NPV	negative predictive value
PCR	polymerase chain reaction
PPV	positive predictive value
