# Supplementary material for: Pre-Analytical Cleanup of Feline Feces Improves DNA Extract Quality, Reduces Post-Extraction PCR Inhibition, and Enhances Molecular Detectability of Intestinal Protozoa
Source: Curr Issues Mol Biol. 2026 Jul 11;48(7):707. doi: 10.3390/cimb48070707 (PMC13408829; doi:10.3390/cimb48070707)
Supplement: Supplementary file 1 [file cimb-48-00707-s001.zip › cimb-4397967-supplementary.pdf]

Table S1. Simplified technical and analytical summary of the in-house one-tube nested real-time PCR-HRM assay used for detection of *Tritrichomonas foetus* in feline fecal DNA extracts.

Parameter	Description / value
**Assay format**	One-tube nested real-time PCR-HRM; closed-tube readout without agarose gel electrophoresis.
**Target and amplicon**	*Tritrichomonas foetus* DNA; partial ITS1-5.8S rRNA region; expected nested amplicon 208 bp.
**Instrument and chemistry**	Gentier 48E real-time PCR system; qPCR-HS Mix EvaGreen chemistry.
**Reaction volume and template input**	20 μL final reaction volume; 2.0 μL DNA template per reaction.
**Reaction composition**	10.0 μL 2× qPCR-HS Mix EvaGreen, 6.0 μL nuclease-free water, 2.0 μL 10× primer mix, and 2.0 μL DNA template.
**Primers**	Outer primers TFR3 and TFR4; inner primers TFITS-F and TFITS-R.
**Final primer concentrations**	TFR3 and TFR4: 12.5 nM each; TFITS-F and TFITS-R: 0.25 μM each.
**Cycling and HRM conditions**	95 °C for 5 min; 20 outer cycles: 95 °C for 15 s and 72 °C for 30 s without fluorescence acquisition; 40 nested real-time cycles: 95 °C for 15 s, 57 °C for 20 s, and 72 °C for 30 s with fluorescence acquisition; HRM from 55 °C to 98 °C with 0.5 °C increments.
**Positive-call rule**	Ct detection plus target-concordant dual-peak HRM profile in both duplicates (2/2 rule). Expected dual-peak profile: low-Tm peak approximately 76-77 °C and high-Tm peak approximately 81-82 °C. Non-concordant duplicate results were retested.
**Analytical sensitivity**	Matrix-matched LoD95 approximately 188 copies/reaction; 44/45 wells positive at this level (97.8%) using Ct detection plus dual-peak HRM concordance.
**Analytical specificity**	No target-concordant cross-reactivity was observed in triplicate testing of non-target templates, including *Giardia duodenalis*, *Cryptosporidium felis*, *Cryptosporidium canis*, *Pentatrichomonas hominis*, *Trichomonas gallinae*, and bacterial isolates.
**Bacterial specificity panel**	Salmonella spp., *Escherichia coli*, *Enterobacter cloacae*, *Klebsiella oxytoca*, *Proteus mirabilis*, *Enterococcus faecium*, *Enterococcus faecalis*, *Streptococcus canis*, and *Streptococcus suis*.
**Matrix blank behavior**	Pooled negative fecal eluate occasionally generated background Ct signals, but these lacked the required dual-peak HRM signature and were classified as nonspecific/negative.
**Carryover assessment**	Checkerboard experiment: 48 wells, including 24 positive-control wells and 24 no-template control wells. No target-concordant dual-peak HRM was observed in NTC wells; carryover events = 0.
**Template input optimization**	Clinical fecal extracts were tested using 1, 2, and 3 μL template inputs and serial dilutions; 2 μL was selected as the routine input condition for inhibitor-rich fecal DNA extracts.
**Clinical agreement**	In 147 feline fecal samples, the one-tube nested real-time PCR-HRM assay showed 100% agreement with gel-based single-tube nested PCR (147/147; Cohen's kappa = 1.00).
**Sequencing confirmation**	All 16 nested-positive clinical amplicons were confirmed by Sanger sequencing as *Tritrichomonas foetus*, with 99.51-100.00% nucleotide identity to reference sequences; GenBank accession numbers PZ404891-PZ404906.
**Interpretative limitation**	The assay summary supports use as an in-house molecular screening method in the present paired workflow study. Results should still be interpreted with the predefined Ct/HRM criteria and appropriate controls, especially in inhibitor-rich fecal matrices.

Note: Ct, cycle threshold; DP, dual peak; HRM, high-resolution melting; LoD95, limit of detection at 95% positivity; NTC, no-template control. This table is a technical supplement, not a full reproduction of the separate validation manuscript.

Table S2. Run-level quality control for the Cryptosporidium 18S spike-recovery experiment.

Run	Spike + water Ct r1	Spike + water Ct r2	Spike + water Ct r3	Spike + water Ct r4	Mean Ct	SD	NTC r1	NTC r2	Spike + negative extract r1	Spike + negative extract r2	QC status
1	27.383	26.971	26.556	26.911	26.955	0.339	No Ct	No Ct	34.633	No Ct	Accepted
2	27.774	27.863	28.115	27.453	27.801	0.273	No Ct	No Ct	No Ct	No Ct	Accepted
3	27.099	28.313	28.557	28.714	28.171	0.733	No Ct	No Ct	No Ct	No Ct	Accepted
4	28.074	28.817	28.036	28.911	28.460	0.469	No Ct	No Ct	No Ct	No Ct	Accepted
5	29.300	28.674	28.360	29.011	28.836	0.408	No Ct	No Ct	No Ct	No Ct	Accepted

Note: NTC, no-template control. No Ct indicates no amplification by the end of cycling. Run-specific spike + water Ct values were used as the no-inhibition reference for ΔCt calculations.

Table S3. Summary of spike-recovery results used for the revised manuscript.

Parameter	Direct extraction	Cleanup-based extraction	Statistical comparison
n paired extracts	44	44	
Mean Ct, median (IQR)	34.51 (33.65-34.85)	30.72 (29.24-32.62)	Wilcoxon p = 1.14e-13
**Δ**Ct inhibition, median (IQR)	6.37 (5.26-6.84)	2.75 (1.35-4.37)	Wilcoxon p = 1.14e-13
Spike recovery %, median (IQR)	1.21 (0.87-2.62)	14.90 (4.83-39.49)	Wilcoxon p = 1.14e-13
Relevant inhibition, **Δ**Ct > 1	44/44	37/44	Exact McNemar p = 0.0156
Strong inhibition, **Δ**Ct > 3	43/44	20/44	Exact McNemar p = 2.38e-07
**Δ**Ct reduction after cleanup, median (IQR)	2.80 (1.33-4.83)		

Note: ΔCt inhibition = max (mean Ct in the presence of fecal DNA extract minus the run-specific mean Ct of spike + water, 0). Spike recovery was calculated as 2^-ΔCt × 100 and capped at 100%. Reactions without amplification were conservatively coded as Ct = 35.0 for calculations. Spearman correlation between A260/230 and ΔCt: direct extracts, rho = -0.495, p = 0.000639; cleanup extracts, rho = -0.179, p = 0.245.

Table S4. Sample-level raw Ct data and DNA quality parameters.

Run	Lp.	Sample ID	Direct A260/230	Cleanup A260/230	Direct Ct r1	Direct Ct r2	Direct mean Ct	Cleanup Ct r1	Cleanup Ct r2	Cleanup mean Ct	Spike + water mean Ct
1	1	149	0.42	0.97	34.911	34.248	34.579	29.240	29.773	29.506	26.955
1	8	164	0.50	1.93	33.613	34.191	33.902	27.910	27.243	27.576	26.955
1	13	190	0.45	2.01	No Ct	No Ct	35.000	33.871	34.220	34.046	26.955
1	19	224	1.10	2.06	34.555	No Ct	34.778	30.817	31.007	30.912	26.955
1	20	230	0.40	1.73	No Ct	No Ct	35.000	29.874	30.119	29.996	26.955
1	22	241	1.00	1.91	No Ct	No Ct	35.000	28.955	29.033	28.994	26.955
1	24	26	0.26	2.39	34.775	No Ct	34.888	33.022	34.070	33.546	26.955
1	26	147	0.53	2.20	34.618	34.220	34.419	29.465	28.008	28.736	26.955
1	28	69	1.54	1.54	33.087	32.812	32.950	28.441	29.775	29.108	26.955
2	29	115	0.59	2.10	34.393	No Ct	34.697	31.465	31.007	31.236	27.801
2	31	304	1.14	1.21	No Ct	34.163	34.581	33.176	32.237	32.707	27.801
2	43	102	0.50	1.20	34.829	33.845	34.337	27.855	27.893	27.874	27.801
2	45	265	0.47	0.54	33.985	No Ct	34.492	31.422	31.581	31.502	27.801
2	47	502	0.25	0.78	33.480	No Ct	34.240	28.286	29.567	28.927	27.801
2	49	512	0.29	0.82	31.666	31.129	31.398	28.418	28.465	28.441	27.801
2	51	569	0.43	1.89	33.315	31.507	32.411	28.324	28.738	28.531	27.801
2	53	609	0.33	1.73	34.597	No Ct	34.799	32.066	32.246	32.156	27.801
2	56	647	1.07	1.75	34.343	No Ct	34.672	30.921	31.553	31.237	27.801
2	58	661	0.22	0.63	34.222	No Ct	34.611	33.770	32.883	33.327	27.801
3	60	164	0.88	1.35	No Ct	No Ct	35.000	34.799	33.971	34.385	28.171
3	61	167	2.03	2.30	32.612	30.338	31.475	28.654	29.952	29.303	28.171
3	64	181	1.08	1.64	No Ct	No Ct	35.000	30.497	29.568	30.032	28.171
3	68	221	1.97	2.03	34.391	No Ct	34.695	29.476	29.097	29.287	28.171
3	73	310	0.58	1.36	33.645	32.444	33.044	30.363	30.707	30.535	28.171
3	77	366	0.53	0.83	No Ct	No Ct	35.000	33.702	32.551	33.127	28.171
3	79	373	1.12	1.87	33.657	No Ct	34.328	28.127	29.079	28.603	28.171
3	80	374	0.49	0.92	No Ct	34.047	34.523	32.108	33.074	32.591	28.171
3	82	419	1.90	2.12	32.951	34.077	33.514	28.606	29.079	28.843	28.171
4	88	461	1.11	1.98	34.702	No Ct	34.851	29.535	28.453	28.994	28.460
4	89	490	1.06	1.30	33.450	34.222	33.836	28.343	30.621	29.482	28.460
4	91	504	1.57	2.14	No Ct	No Ct	35.000	30.025	30.419	30.222	28.460
4	94	576	0.83	1.44	No Ct	33.752	34.376	30.902	29.313	30.108	28.460
4	95	577	0.98	1.49	31.011	32.151	31.581	31.677	31.159	31.418	28.460
4	97	104	1.71	2.26	33.420	32.025	32.722	29.791	30.622	30.206	28.460
4	98	111	2.24	2.33	32.211	31.734	31.973	30.701	29.052	29.877	28.460
5	3	573	0.66	1.21	No Ct	No Ct	35.000	No Ct	33.477	34.239	28.836
5	6	520	2.14	2.20	31.025	32.477	31.751	31.588	31.509	31.549	28.836
5	16	214	0.78	1.24	No Ct	33.713	34.356	31.589	32.703	32.146	28.836
5	34	605	1.19	1.87	33.301	34.078	33.690	33.137	33.854	33.495	28.836

5	37	20	2.26	2.10	30.707	No Ct	32.853	31.227	32.008	31.617	28.836
5	40	515	2.10	1.17	33.117	34.578	33.847	33.412	32.097	32.755	28.836
5	84	428	1.89	2.01	34.714	No Ct	34.857	33.713	No Ct	34.356	28.836
5	100	118	0.80	1.38	No Ct	34.367	34.683	31.855	32.723	32.289	28.836
5	101	11	0.80	1.60	No Ct	No Ct	35.000	No Ct	33.119	34.059	28.836

Note: Raw No Ct results were coded as Ct = 35.0 only for calculating mean Ct, ΔCt, and spike recovery.

Table S5. Sample-level derived inhibition and spike-recovery estimates.

Run	Lp.	Sample ID	Direct ΔCt	Cleanup ΔCt	ΔCt reduction	Direct recovery %	Cleanup recovery %	Direct class	Cleanup class	Direct ΔCt >3	Cleanup ΔCt >3
1	1	149	7.624	2.551	5.073	0.51	17.06	Strong	Moderate	Yes	No
1	8	164	6.947	0.621	6.326	0.81	65.01	Strong	Mild	Yes	No
1	13	190	8.045	7.090	0.954	0.38	0.73	Strong	Strong	Yes	Yes
1	19	224	7.822	3.957	3.866	0.44	6.44	Strong	Strong	Yes	Yes
1	20	230	8.045	3.041	5.004	0.38	12.15	Strong	Strong	Yes	Yes
1	22	241	8.045	2.039	6.006	0.38	24.34	Strong	Moderate	Yes	No
1	24	26	7.932	6.591	1.342	0.41	1.04	Strong	Strong	Yes	Yes
1	26	147	7.464	1.781	5.682	0.57	29.09	Strong	Moderate	Yes	No
1	28	69	5.994	2.153	3.842	1.57	22.49	Strong	Moderate	Yes	No
2	29	115	6.895	3.435	3.460	0.84	9.25	Strong	Strong	Yes	Yes
2	31	304	6.780	4.905	1.875	0.91	3.34	Strong	Strong	Yes	Yes
2	43	102	6.536	0.073	6.463	1.08	95.08	Strong	No/low	Yes	No
2	45	265	6.691	3.700	2.991	0.97	7.69	Strong	Strong	Yes	Yes
2	47	502	6.439	1.125	5.313	1.15	45.84	Strong	Moderate	Yes	No
2	49	512	3.596	0.640	2.956	8.27	64.16	Strong	Mild	Yes	No
2	51	569	4.610	0.730	3.880	4.10	60.30	Strong	Mild	Yes	No
2	53	609	6.997	4.355	2.642	0.78	4.89	Strong	Strong	Yes	Yes
2	56	647	6.870	3.436	3.434	0.85	9.24	Strong	Strong	Yes	Yes
2	58	661	6.810	5.525	1.285	0.89	2.17	Strong	Strong	Yes	Yes
3	60	164	6.829	6.214	0.615	0.88	1.35	Strong	Strong	Yes	Yes
3	61	167	3.304	1.132	2.172	10.12	45.62	Strong	Moderate	Yes	No
3	64	181	6.829	1.862	4.968	0.88	27.51	Strong	Moderate	Yes	No
3	68	221	6.525	1.116	5.409	1.09	46.15	Strong	Moderate	Yes	No
3	73	310	4.874	2.364	2.509	3.41	19.42	Strong	Moderate	Yes	No
3	77	366	6.829	4.956	1.873	0.88	3.22	Strong	Strong	Yes	Yes
3	79	373	6.158	0.432	5.725	1.40	74.11	Strong	No/low	Yes	No
3	80	374	6.353	4.420	1.933	1.22	4.67	Strong	Strong	Yes	Yes
3	82	419	5.343	0.672	4.671	2.46	62.77	Strong	Mild	Yes	No
4	88	461	6.391	0.534	5.857	1.19	69.04	Strong	Mild	Yes	No
4	89	490	5.376	1.022	4.354	2.41	49.23	Strong	Moderate	Yes	No
4	91	504	6.540	1.762	4.778	1.07	29.47	Strong	Moderate	Yes	No
4	94	576	5.917	1.648	4.269	1.66	31.91	Strong	Moderate	Yes	No
4	95	577	3.122	2.958	0.163	11.49	12.86	Strong	Moderate	Yes	No
4	97	104	4.263	1.747	2.516	5.21	29.79	Strong	Moderate	Yes	No
4	98	111	3.513	1.417	2.096	8.76	37.45	Strong	Moderate	Yes	No
5	3	573	6.164	5.402	0.761	1.39	2.36	Strong	Strong	Yes	Yes
5	6	520	2.915	2.712	0.202	13.26	15.26	Moderate	Moderate	No	No
5	16	214	5.520	3.310	2.210	2.18	10.08	Strong	Strong	Yes	Yes
5	34	605	4.853	4.659	0.194	3.46	3.96	Strong	Strong	Yes	Yes

5	37	20	4.017	2.781	1.236	6.18	14.55	Strong	Moderate	Yes	No
5	40	515	5.011	3.918	1.093	3.10	6.61	Strong	Strong	Yes	Yes
5	84	428	6.021	5.520	0.501	1.54	2.18	Strong	Strong	Yes	Yes
5	100	118	5.847	3.453	2.394	1.74	9.13	Strong	Strong	Yes	Yes
5	101	11	6.164	5.223	0.941	1.39	2.68	Strong	Strong	Yes	Yes

Inhibition class: no/low, ΔCt <= 0.5; mild, >0.5 to 1.0; moderate, >1.0 to 3.0; strong, >3.0.
